# Effect of Seawall Embankment Reclamation on the Distribution of Cr, Cu, Pb and Zn Pollution in Invasive *Spartina alterniflora* and Native *Phragmites australis* Coastal Saltmarshes of East China

**DOI:** 10.3390/biology12020253

**Published:** 2023-02-06

**Authors:** Jian Li, Zhanrui Leng, Hui Jia, Lili Wei, Taitiya Kenneth Yuguda, Daolin Du

**Affiliations:** 1 School of Emergency Management, School of the Environment and Safety Engineering, Jiangsu University, Zhenjiang 212013, China; 2 State Key Laboratory of Marine Environmental Science, Xiamen University, Xiamen 361102, China; 3 Institute of Urban Environment, Chinese Academy of Sciences, Xiamen 361021, China

**Keywords:** coastal reclamation, coastal embankment, invasive species, saltmarsh, alien plant invasion, biogeochemical cycling, pollution

## Abstract

**Simple Summary:**

Coastal reclamation is the process of converting sea area to land. It is a land use practice commonly associated with loss and damage to the environment, pollution, and affecting the subsistence of man, animals, and plant life. In China, seawalls are constructed along the coastlines to transform wetlands into additional productive land. However, the construction of these seawalls comes with some negative environmental impacts. This study examined the impact of seawalls on the distribution of trace metal (TM) pollution in reclaimed and unreclaimed wetlands. Plant and soil samples were collected from the affected wetlands and analyzed in the laboratory. Results show that the construction of the seawalls, coupled with factors such as plant organic matter, soil water, and salt content influenced the significant increase in TM pollution in the reclaimed wetlands compared to the unreclaimed wetlands. A comparison of the results with international standard quality guidelines shows that the soils from wetlands on the coastline of the study region were moderate to severely polluted by copper, zinc, and chromium. Overall, soils affected by reclamation posed a greater ecological risk than in unreclaimed wetlands. Stakeholders can use this scientific outcome to mitigate TM pollution caused by seawall construction in coastal ecosystems.

**Abstract:**

Coastal reclamation by seawall embankments and the spread of invasive C_4_ perennial grass *Spartina alterniflora* have recently become more prevalent in eastern China’s coastal wetlands. While trace metals (TMs), carbon, and nitrogen dynamics concerning reclamation have extensively been explored across China’s coastal wetlands, to date, the impact of reclamation by coastal embankment and exotic plant invasion on TMs’ pollution dynamics in coastal marshes remains largely unexplored. We compared TMs Cr, Cu, Pb, and Zn cumulation in coastal embankment-reclaimed versus unreclaimed *S. alterniflora* and *Phragmites australis* saltmarshes in eastern China coastal wetlands. In both *S. alterniflora* and *P. australis* marshes, coastal embankment reclamation spurred an increase in Cr, Cu, Pb, and Zn concentrations by 31.66%, 53.85%, 32.14%, 33.96% and by 59.18%, 87.50%, 55.55%, 36.84%, respectively, in both marsh types. Reclamation also reduced plant biomass, soil moisture, and soil salinity in both plants’ marshes. Our findings suggest that the impact of coastal embankment reclamation and replacement of native saltmarshes by invasive *S. alterniflora* had a synergistic effect on TM accumulation in the *P. australis* marshes, as corroborated by bioaccumulation and translocation factors. Reclamation by coastal embankments and invasive alien plants could significantly impair the physico-chemical properties of native plant saltmarsh and essentially weaken the accumulation of Cr, Cu, Pb, and Zn potential of the coastal saltmarshes. Our findings provide policymakers with an enhanced knowledge of the relationship between reclamation, plant invasiveness, and TM pollution dynamics in coastal wetlands, providing a baseline for attaining future goals and strategies related to the tradeoffs of various wetland reclamation types.

## 1. Introduction

Coastal wetlands, despite their critical ecological functions, are among the most endangered ecosystems, with significant alterations related to human activities. [1,2]. Invasive alien plant species (IAPS) and coastal embankments have been introduced imported and spread throughout critical coastal habitats worldwide to restore and preserve these deteriorated coastal ecosystems in recent times [3,4]. Globally, coastlines have undergone significant modification, necessitating the erection of seawalls, which have been essential since ancient times, to act as coastal protection. [4]. Seawall embankments, which have traditionally been used for coastal fortifications such as flood mitigation and safeguarding settlements from the influence of waves and tides, are now progressively deployed to enclose coastal wetlands for agricultural and industrial purposes [4,5,6]. In China, reclamation activities have led to the deployment of thousands of kilometers of seawalls along China’s coastlines to prevent the threat of *S. alterniflora* invasion and to transform native intertidal habitats into additional productive land [7]. However, despite its ecological benefits, *S. alterniflora* invasion in China has coincided with reclamation by embankment seawalls for wetlands restoration. As a result, coastal reclamation and invasive alien species are now the leading causes of coastal wetlands degradation, threatening the health and functioning of ecosystems [7,8].

Anthropogenic impacts of wetland reclamation, particularly soil disturbance, are connected to decreases in soil organic carbon (SOC) reserves [9], ecological deterioration, trace metal (TM) contamination, and eutrophication [10,11,12]. The rapid expansion of China’s coastline areas has coincided with a significant environmental problem of TM contamination, partly related to coastal reclamation for agriculture and aquaculture [13,14,15]. Similarly, various reclamation types have been found to considerably increase overall TMs and pollution levels, with varying effects depending on native or invasive plant dominance in wetlands marshes [16,17,18,19]. Presently, the reclamation of wetlands in South China has resulted in significant TM pollution concerns [20,21]. Reclamation activities have influenced the composition of TM fractions, particularly Cd, Pb, Zn, and Ni, making residual fractions the predominant biogeochemical component for the metals identified [22]. Following intensive coastal reclamation, changes in soil organic carbon and nitrogen pools have been observed in various reclaimed regions. Findings imply that coastal reclamation alters the carbon and nitrogen sinks of coastal wetlands by altering the amount, structure, and dynamics of SOC and soil organic nitrogen (SON) pools after the transformation of native wetlands to other land uses. As a result of coastal reclamation, changes in the quantity and quality of extrinsic substances reverting to the soil, as well as soil physiochemical characteristics, were all contributing variables [3,23,24,25,26,27].

Coastal embankment has been proven to alter wetland ecosystem composition and functioning. Although coastal embankment enhanced flora growth, it negatively impacted subsurface saline water extraction [28], whereas reduced soil salinity accompanying coastal embankments was the main driver of changes in plant and soil nitrogen pools [29]. Due to coastal embankments, soil microbial ecosystems are altered by nutrient substrates modification and the lack of seawater [30], whereas copper phytoavailability is facilitated by luminous soluble organic materials in coastal wetlands altered by coastal embankment [31]. However, in mangrove forests, the impacts of embankments on wrack composition and dynamics may be lower than on unvegetated coastlines [32]. The overall effect of coastal embankment reclamation on intertidal ecosystems could result in the accumulation of tens of thousands of tonnes of sediment, burying hundreds of tonnes of carbon and nitrogen, as well as several tonnes of TMs [33,34].

Reclamation of an invasive plant-dominated salt marsh by embankment seawalls has been proven to significantly alter soil carbon, nitrogen, salinity, litter, water content, and plant biomass outcomes [3,24,25,26,27]. SOC and grain size were found to be important factors in influencing the distribution of TMs in a long-term reclaimed area of a China River Delta following an investigation of TMs and soil physicochemical characteristics [35]. Therefore, we hypothesize that reclamation by coastal embankment may alter the potential of invasive *S. alterniflora* and native Phragmites australis saltmarshes to accumulate TM dynamics. The primary objectives of this study were to (i) evaluate the impact of the coastal embankment on TMs pollution in both reclaimed and unreclaimed saltmarshes; (ii) ascertain whether reclamation by coastal embankment and invasive *S. alterniflora* have synergistic effects on TM accumulation in the coastal saltmarshes.

## 2. Materials and Methods

### 2.1. Study Area

**Figure 1:**
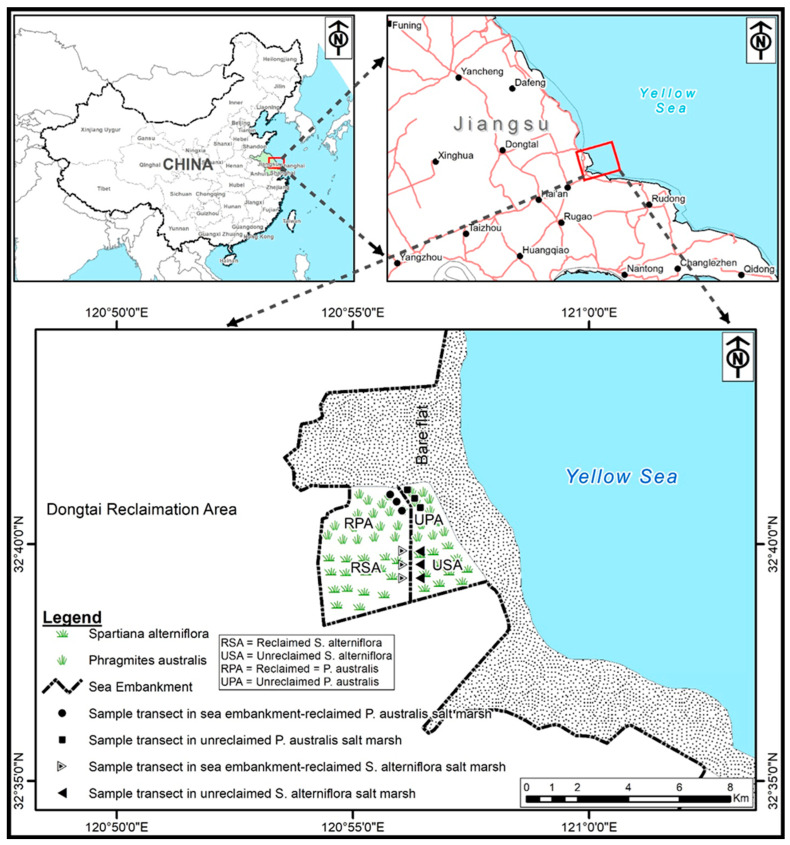
Location in Dongtai, Jiangsu, where samples were obtained.

The study site (Figure 1) is situated close to the Yellow Sea coast in Dongtai, Jiangsu Province, with an average yearly temperature of 15 °C, a mean temperature range of −0.3 to 1.3 °C, and a minimum temperature of 17.3 °C. The average sunshine is 2199–2362 h, with solar radiation averaging 116.2 × 4.184–121.0 × 4.184 kJ cm^−2^. In July, the average temperature is 26.7–27.4 °C, with the maximum temperature hitting 39 °C. The mean annual precipitation ranges from 980 to 1070 mm, a mean of 1061 mm, with May and July contributing to almost 70% of the overall. Severe weather includes tornados, thunderstorms, hailstorms, cyclones, storm surges, and fog, to name a few. The undersea intertidal period can last anywhere from 7 to 12 h. The storm surge is 1.27–4.61 m. With a pH of roughly 8, the salinity is 2.953–3.224 % [36]. Mudflats at the intertidal zone and marshlands at mid and high tides make up the coastal wetlands of Dongtai [37]. The broad area of *S. alterniflora* spread has risen considerably since its introduction. By 1992, the plot began 3 km away from the Dongtai–Dafeng border and spread into the Dafeng tideland. According to the 1.8 m elevation of the coastal marsh boundary, *S. alterniflora* wetlands have extended about 1 km seaward, with altitudes varying from 3.5 to 1.8 m. The marshland, which was 1.1 km wide and made up 70% of the land, was determined to have optimal growth at heights of 2.0–2.5 m [38]. Of a total span of 15,339 km, Dongtai administration built a 6163 km coastal dyke to avoid the threat of *S. alterniflora* invasion. When it was enclosed, the soils and native flora were in fair condition. *Phragmites australis*, *Suaeda salsa*, *Spartina alterniftora*, and *Imperata cylindrica var.* covered approximately 80% of the soil [38]. At the time of enclosure, the soil and natural flora were in good condition. Almost 80% of the ground was occupied by invasive and native wetland plants [38].

### 2.2. Field Sample Collection

#### Biomass and Sediment Sampling

In July 2022, plant biomass was sampled in three 50 cm × 50 cm transects in each segment of the coastal embankment-reclaimed *Spartina alterniflora* (RSA), coastal embankment-reclaimed *Phragmites australis* RPA, unreclaimed *Spartina alterniflora* (USA), and unreclaimed *Phragmites australis* UPA saltmarshes. Three soil blocks (10 cm by 10 cm by 60 cm depth) were excavated to obtain plant roots. The coastal embankment in the study region infringed on mudflats for a substantial period, according to the analyses of Thematic Mapper landsat imagery [38]. Therefore, plant density and height were recorded on the spot. Samples of *S. alterniflora* and *P. australis* parts, which comprise aboveground biomass AGB (leaf, branches, and stem), belowground biomass BGB (roots), and litter biomass LB (litterfall), were gathered apart. The BGB measure plots were 1 m × 1 m in dimension, with the depth varying depending on the plant root depth. *P. australis* roots are typically found at depths of 60 cm or less, whereas other species’ roots are located at shallower depths [39]. Each plot was excavated for soil and filtered through a fine screen to extract all of the roots, with the LB encompassing the whole litter mass inside the plot. A balance (Model 4504 M P, Sartorius Corp., Edge-wood, Data Weighing Systems, Inc., IL, USA) was used to determine the fresh weight of each transect’s AGB, BGB, and LB portions. Using a (PVC) corer to obtain sediment samples, triplicate sediment cores were taken in each transect to a depth of 30 cm and layered at 10 cm increments (i.e., 0–10 cm, 10–20 cm, and 20–30 cm). A total of 36 sediment samples were collected, and enclosed in polyethylene plastic bags, before being sent to the laboratory for analysis.

### 2.3. Laboratory Analyses

Following filtration and thorough washing of excavated soil with water, residual roots in the 100-mesh sieve, as well as other plant components, were thoroughly cleaned and oven-dried at 65 °C to determine leaf, stem, litter, root, and total biomass [40]. Original soil subsamples were weighed and oven-dried at 105 °C to obtain the bulk density using the cutting ring method [24]. Floral and faunal remains were meticulously extracted from soil samples and separated into multiple subsamples following proper mixing. To measure soil pH, salinity, soil carbon, and nitrogen, each subsample was subjected to air drying and filtration through 1 mm sieves. With a 1:2.5 soil-to-water concentration, a glass membrane electrode was employed to measure the pH of the soil. The soil salinity was determined using a conductivity meter; a soil-to-water ratio of 1:5 (Yang et al., 2013). The dichromate oxidation method was used to determine the amount of organic matter in the soil [41].

To determine the soil water content, plant and organic debris in the soil samples were separated with forceps, 2 mm sieved, then oven-dried to a consistent weight at 50 °C. First, the dry weight of the entire sample range was divided by the volume of the core section to obtain the bulk density of each sample. Next, the carbon and nitrogen concentrations were determined using an elemental analysis (Vario EL III elemental analyzer. Elementar, Hesse, Germany). Each component’s carbon stock was determined depending on the previously obtained carbon or nitrogen content and biomass values.

### 2.4. Trace Metal Determination

After both saltmarsh biomass samples were cleaned with deionized water to remove any dust, 0.2 g of marsh sample taken was dissolved using vigorous nitric acid (HNO_3_). Upon drying and mixing, all plant parts samples were split into 0.5 g sub-samples due to differences in *S. alterniflora* and *P. australis* between sample plots. Flame atomic absorption spectrometry (AAnalyst 800, PerkinElmer, Waltham, MA, USA) was used to detect Cu, Zn, Pb, and Cr. Cd, Cr, Cu, and Zn were analyzed using the flame atomic absorption method, whereas Pb was analyzed using the graphite furnace atomic absorption technique.

#### 2.4.1. Bio-Concentration and Translocation Factors

The bio-concentration factors for the TMs analyzed in this study were computed using the following formulae:

(1) $$BCF_{leaf}=C_{leaf}/C_{sediment}$$

(2) $$BCF_{stem}=C_{stem}/C_{sediment}$$

(3) $$BCF_{root\;}=C_{root}/C_{sediment}$$

where *C_leaf_, C_stem_*, and *C_root_* represent the concentrations of metal in the leaf, stem, and root, respectively, and *C_sediment_* is the concentration of metal in the sediment.

The preceding formulae can also be used to compute the translocation factors (TF) of the TMs examined:

(4) $$TF_{leaf}=C_{leaf}/C_{root}$$

(5) $$TF_{stem}=C_{stem}/C_{root}$$

where *C_leaf_, C_stem_*, and *C_root_* represent the concentrations of metal in the leaf, stem, and root, respectively. This study estimated and provided average TF values for AGB (leaf and stem).

#### 2.4.2. Geoaccumulation Index

The geoaccumulation index (*I_geo_*), which assesses soil pollution, could estimate wetland sediment pollution. It is expressed as:

(6) Igeo=log2Cn1.5Bn

where *C_n_* and *B_n_* stand for the studied TM concentration and the TMs’ geological baseline value of element *n*, respectively, and 1.5 is the lithogenic effects-related baseline matrix adjustment factor. While the *I_geo_* guidelines refer to [42], the geologic baseline values for the observed TMs in the Dongtai estuary are presented in [43].

### 2.5. Statistical Analysis

Variations in plant characteristics and the effects of reclamation on soil moisture, bulk density, pH, salinity, TC, TN, AGB, and BGB were all statistically examined for each community. The implications of community structure, restoration, and depth, as well as their interactions, on the physicochemical properties of the soil, as well as varying fractions of TM concentrations between seawall reclaimed and unreclaimed marshes were determined using analysis of variance ANOVA and Tukey HSD multiple comparisons. To determine the correlations between the targeted TMs and the chosen soil variables, redundancy analysis (RDA) was utilized. SPSS version 26.0 was used for all data analysis. Principal component analysis (PCA) was employed to elucidate the correlations between multiple variables, and Origin 9.8 was used to produce graphical representations of the data.

## 3. Results

### 3.1. Plant Biological Characteristics and Biomass Contents

**Figure 2:**
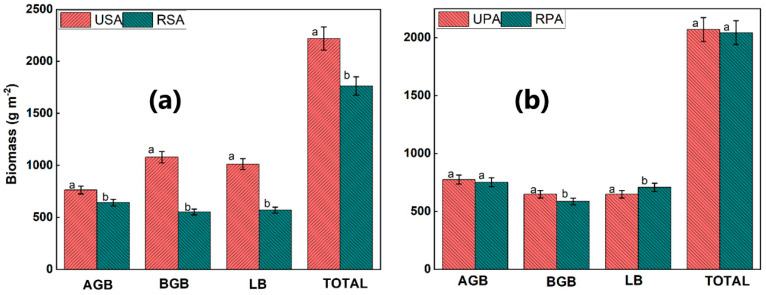
(**a**) accumulation of plant biomass in the *S. alteniflora* saltmarsh (**b**) accumulation of plant biomass in the *P. australis* saltmarsh, Error bars indicate mean ± standard error (*n* = 3). USA = unreclaimed *S. alteniflora* saltmarsh; RSA = reclaimed *S. alteniflora* saltmarsh; UPA = unreclaimed *P. australis* saltmarsh; RPA = reclaimed *P. australis* saltmarsh.

Total biomass, including leaf, stem, litter, and root, was considerably lower in the RSA marsh than in the USA marsh. *S. alterniflora* grew taller than *P. australis* (F = 20.6, p = 0.001) and had much-enhanced plant density (F = 11.5, *p* < 0.001). In the RPA salt marsh, root biomass in the (0–30 cm) depth and stem biomass were considerably less than those of the UPA salt marsh. The RSA salt marsh had considerably lower AGB and total biomass than the USA salt marsh (Figure 2a,b). In contrast, LB was significantly higher in the RPA salt marsh compared to the UPA, whereas the statistics of leaf biomass and total biomass did not differ significantly between the two saltmarshes (Figure 2b). Collectively, the USA marsh contained the highest concentrations of AGB, BGB, and LB.

### 3.2. Soil Physicochemical Properties

The coastal embankment RSA saltmarsh had much lower soil moisture and salinity (0–30 cm depth) than the USA salt marsh, as shown in Table 1. The soil pH varied significantly between the RSA and USA salt marsh, with the RSA having the highest pH of both marshes (Table 1). In both saltmarshes, reclamation by coastal embankment did not affect the bulk density of the soil (20–30 cm depth). Excluding SOM (10–30 cm) and BD in the (0–10 cm) soil depth (Table 1), no notable variations were observed in soil pH or salinity between the UPA and RPA saltmarshes.

### 3.3. Soil Trace Metal Pools

**Figure 3:**
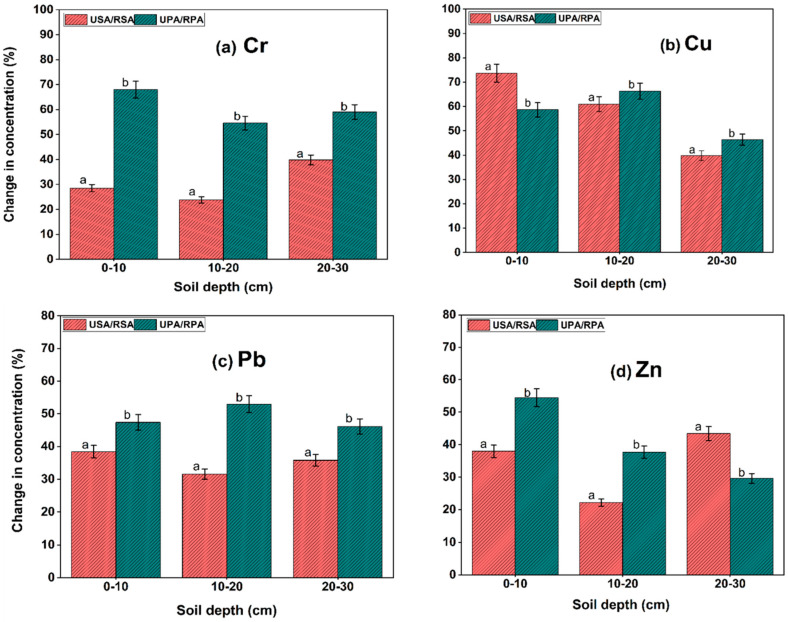
Changes in concentration of trace metals (TMs) (**a**–**d**) due to coastal embankment in both plant marshes. Lower-case letters over the bars denote statistical significance at *p* < 0.05 between the unreclaimed and coastal embankment-reclaimed saltmarshes in the same zone and soil depth.

Reclamation by coastal embankment significantly affected Cr, Cu, Pb, Zn distribution. Overall, TM concentrations were considerably higher in the coastal embankment RSA plant marshes than in the USA (Table 2). The TM accumulation in the USA and RSA saltmarshes ranged from 12.32 to 112.65 mg kg^−1^, respectively, whereas in the *P. australis* saltmarshes, concentrations ranged from 5.25 to 68.71 mg kg^−1^, (Table 2). Cumulation of Cr, Cu, Pb, and Zn in the (0–30 cm) soil depth of the coastal embankment RSA salt marsh increased by 31.66%, 53.85%, 32.14%, and 33.96%, respectively, compared to the USA salt marsh. A similar trend was observed in the soil (0–30 cm) depth of coastal embankment RPA marsh, which increased TM concentrations by 59.18%, 87.50%, 55.55%, and 36.84%, respectively, correlative to the UPA salt marsh as shown in Figure 3. Ultimately, coastal embankment reclamation significantly influenced Cu cumulation in both reclamation types and Cr and Pb cumulation in the *P. australis* marsh. Generally, the concentration of TMs increased steadily with depth, with significant cumulation of Cr, Cu, Pb, and Zn occurring at 20–30 cm depth in both types of marsh, although metal deposition in deeper sediments can be influenced by metal movement and soil conditions. The increased levels of TMs found in the deeper soils of all marsh types revealed, however, that tillage had led to TM loss in the top soils of coastal wetlands because of agricultural runoff and invasive plant removal [44].

### 3.4. Evaluation of Trace Metal Pollution Using Sediment Quality Guidelines (SQGs)

By comparing metal concentrations in soils to those developed by US EPA, CB TECH, NOAA ERL, SQAV TEL, SQO Netherlands, the Ministry of Environment and Energy, Ontario, Canada, Hong Kong (ISQVs), and soil quality thresholds (SQTs) of China SQGs, the degree of TMs was evaluated as shown in Table 3. The CB TECH and SQAV guidelines indicated that all soil samples in the (20–30 cm) depth of the RSA saltmarshes exceeded the threshold for Cr and Cu, and NOAA and US EPA also indicated the exceedance of Cr concentrations. Zn indicated moderate pollution in similar depth according to US EPA. For Pb and Zn, their metal concentrations were below severe effect level except for Zn in the (20–30 cm) depth. In the reclaimed saltmarshes, 70% of the soil samples exceeded the lowest effect level for Cr, Cu, and Pb. These showed that a negative toxicological impact on the environment of the marsh might be anticipated. But almost all samples fell short of the severe effect threshold (SEL), over which the bulk of soil-dwelling microorganisms are anticipated to suffer substantially negative impacts for Cr and Cu, except that 30% (unreclaimed) and ~60 % (reclaimed) soil samples for Cr and Cu could have detrimental effects on benthic organisms due to their concentrations. In particular, we discovered that reclamation increased Cr and Zn enrichment by ~40% compared to non-reclamation, indicating that Cr and Zn are metal elements that need to be taken into consideration in subsequent reclamation [22]. According to the standards for sediment quality established by NOAA, the US EPA, CHINA SEPA, and SQO Netherlands, Cr and Cu were both moderately and extensively polluted metal elements, respectively, and their amounts were expected to periodically impact the saltmarshes negatively. These prevailing opinion sediment quality recommendations and the comparative study of the TM levels in saltmarsh soils indicated that metals (Cr, Cu, and Pb) in the mudflats posed a significant or intermediate ecological threat to the existing reclamation. The increase in Cu content may have been driven by long-term agronomic and aquaculture practices [45], whereas the main sources of Cr may have been domestic sewage disposal and industrialization [46].

Table 4 shows comparisons of the mean heavy metal content with that in other locations. The mean content of Cr for this study of 112.6 mg kg^−1^ was higher than those in other regions of China (51.4–68.3 mg kg^−1^) [22,55,56,57] and Malaysia (2.68 mg kg^−1^) [58], but lower than that obtained in Turkey (163.0 mg kg^−1^) [59]. The mean content of Cu was similar to that of the Yangtze River Estuary [55] and the Kavak Delta in Turkey [59], but significantly lower than that of the Pearl River Delta, China (70.0 mg kg^−1^) [57] and significantly higher than in Yangtze River Delta, China [22]. For Zn, concentrations were on par with those of the other studies, except for the Langat River, Malaysia which had a relatively low concentration of Zn (29.7 mg kg^−1^). However, the concentration of Pb (32.3 mg kg^−1^) was higher in most of the other regions, except for the Pearl River Delta which had a Pb concentration of (48.3 mg kg^−1^) [57]. This shows that the levels of Cr and Pb in the saltmarsh soils of Jiangsu’s Dongtai Estuary are relatively problematic and need to be addressed.

### 3.5. Geoaccumulation Index

**Figure 4:**
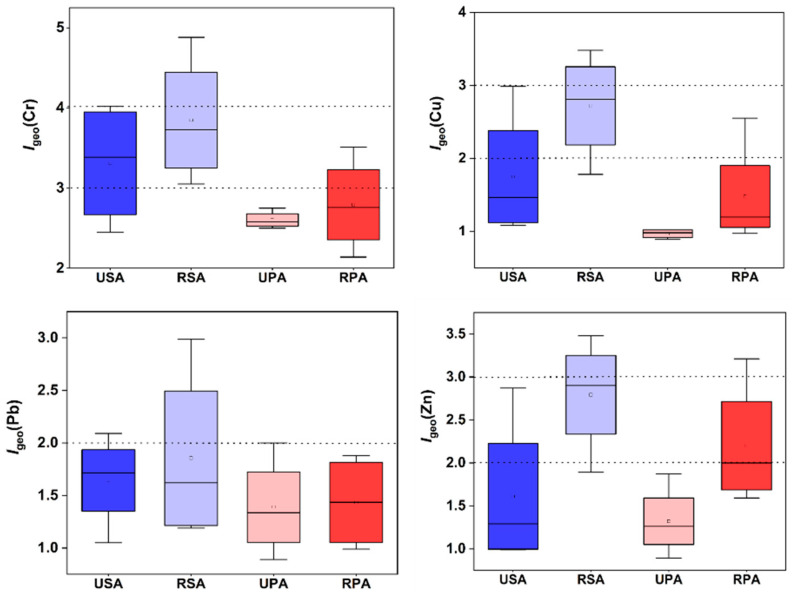
The I_geo_ values for TMs in reclaimed and unreclaimed soils of *S. alterniflora* and *P. australis* saltmarshes.

Figure 4 displays the computed Igeo values for TMs in the various plant marshes’ reclaimed and unreclaimed soils. Because the I_geo_ index of the reclaimed soils of the *S. alterniflora* marsh was greater than two, Cr, Cu, and Zn were classified as “polluted”, whereas the unreclaimed soils’ I_geo_ values indicated that all soils were “unpolluted to moderately polluted”, with the exception of Cu and Pb. Soils from reclaimed *P. australis* were “moderately polluted” with Zn and Cr. Comparatively, the I_geo_ values for Cr, Cu, Pb, and Zn in the reclaimed soils were above 1, but in the unreclaimed *P. australis* soils, Cu, Pb, and Zn had values below one, indicating an “unpolluted” state in some depths, although Cr seemed to have “moderately contaminated” the soils. In general, Cr contamination was found in all of the marsh soil samples. According to I_geo_ estimates for Cr, reclaimed soils were “moderately to highly contaminated”. The Cr I_geo_ values in RSA soils ranged from 3 to 5, suggesting a seriously contaminated status [60]. The I_geo_ results for Cr in the soils of the USA, UPA, and RPA specifically indicated that they were “moderately to highly contaminated.” Overall, the computed values of I_geo_ for Cr, Cu, Pb, and Zn ranged from 2.5 to 5.00, 0.84 to 3.63, 0.86 to 2.99, and 0.84 to 3.63, respectively. According to the quantitative data analysis of the I_geo_, both reclamation soils were enriched in Cr, which has significant negative effects on marine, terrestrial, and plant life. In contrast, the RSA soils were only moderately polluted with Cu and Zn.

### 3.6. Bio-Concentration and Translocation Factors

Our findings showed that different types of reclamation and parts of the biomass from *S. alterniflora* and *P. australis* had varying amounts of TM cumulation (Table 5). TM concentrations in plants were rather high and were found in the following order: Zn > Cr > Cu > Pb. The highest Zn levels in *S. alterniflora* plants are related to the high Zn levels in the nearby soils. In the RSA marshes, Cr concentrations were likewise noticeably greater than those of Cu and Pb. The fact that Zn and Cr are crucial trace elements for plants may help to explain the high amounts of these two metals [60].

**Figure 5:**
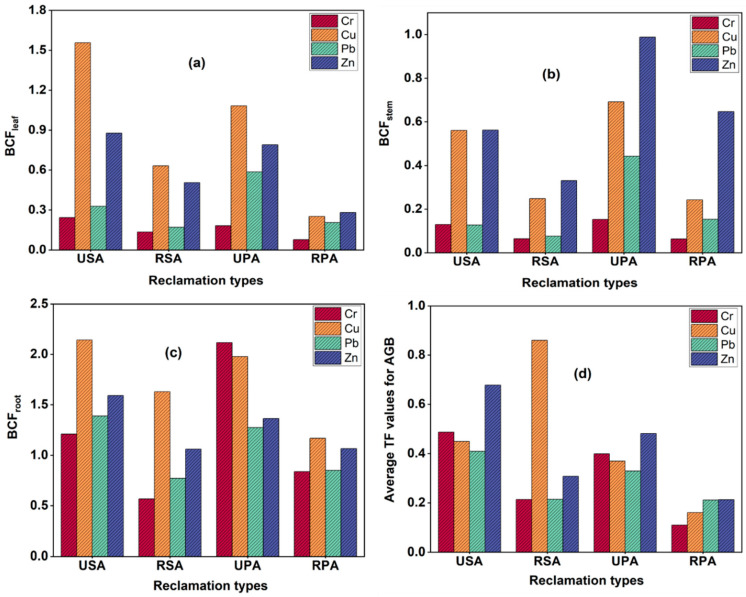
Bio-concentration factors (BCF) for the metals in leaf (**a**), stem (**b**), and root (**c**) and the average translocation factors (TF) for the metals in AGB of marsh plants (**d**).

The bio-concentration factor (BCF), which measures the ratio of metal concentrations in plant tissue to that in soil, can evaluate the capacity of aquatic and plant organisms to uptake contaminants from soil [60,61]. According to findings, the leaf BCF values of Cr, Cu, Pb, and Zn in the unreclaimed plant marshes range from 0.18–0.24, 1.08–1.56, 0.32–0.59, and 0.78–0.87, respectively. The stem BCF values range from 0.13–0.15, 0.56–0.69, 0.12–0.44, and 0.56–0.99, and the root BCF values range from 1.21–2.12, 1.98–2.14, 1.27–1.39, and 1.36–1.59, respectively. In the seawall embankment-reclaimed marshes, the leaf BCF values of Cr, Cu, Pb, and Zn range from 0.077–0.13, 0.25–0.63, 0.17–0.20, and 0.28–0.51, respectively. Stem BCF values range from 0.063–0.065, 0.24–0.25, 0.077–0.153, and 0.33–0.65, and the root BCF values range from 0.56–0.84, 1.17–1.63, 0.77–0.85, and 1.06–1.66, respectively (Figure 5).

BCF values less than one (BCF < 1) suggest that *S. alterniflora* and *P. australis* were highly inefficient for TM bioaccumulation, or the presence of the reclamation embankment altered their ability to accumulate TMs. Both unreclaimed marshes exhibited (BCF > 1) values for Cr, Cu, Pb, and Zn in the root, demonstrating that these marshes have a higher bio-accumulation of TMs and are more flexible than embankment-restored wetlands. Figure 5d shows the mean TF values for AGB components. The average values of TF in all reclamation marsh types were substantially less than one (TF < 1), except for Cu in RSA and Zn in the USA, which had TFs of 0.86 and 0.68, respectively. Typically, plant species with TF values greater than one (TF > 1) are considered highly efficient for the translocation of metals from BGB to the AGB of the marsh plant species [60].

## 4. Discussion

### 4.1. Relationships between Trace Metals and Selected Soil Properties

**Figure 6:**
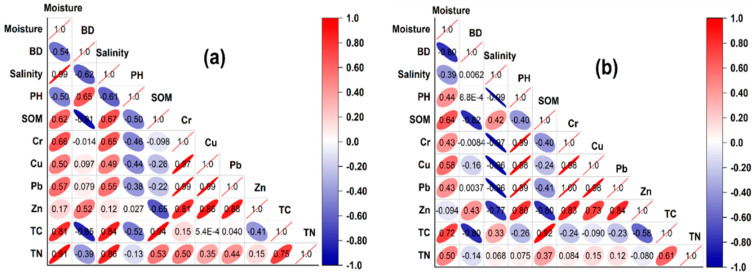
Correlation analysis of TMs, Carbon and Nitrogen fractions, soil physical and chemical properties across communities in the (**a**) unreclaimed and (**b**) seawall-reclaimed saltmarshes.

The use of multivariate analysis, which includes principal component analysis (PCA) and correlation matrix (CM), is a practical approach to identifying TM sources [57,60]. Figure 6 demonstrates the association between TMs and various soil parameters in reclaimed and unreclaimed saltmarshes. The unreclaimed and embankment-reclaimed marshes showed a negative correlation between soil organic matter (SOM) and Cr, Cu, Pb, and Zn. Because of its effective complexing potential for metallic pollutants, some studies [44,62] have demonstrated that SOM can operate as a significant sink for TMs (i.e., Cd, Cr, Cu, Ni, Pb, and Zn). The results of this study appear to support the opposite hypothesis, as Cr, Cu, Pb, and Zn all showed negative associations with SOM, indicating that SOM may have been mostly imported from an outside source into the study area. Zn was significantly correlated with SOM in both forms of reclamation (*p* < 0.01), and it was higher in the embankment-reclaimed marshes.

According to [63], a decrease in soil pH may result in an increase in the availability of TM due to proton competition between the metals and a decrease in negative binding sites. These outcomes also concurred with findings by [64] that SOM could lessen the availability of TM by generating stable complexes. Positive correlations between Cr, Cu, Pb, Zn, and salinity in the unreclaimed saltmarshes indicate that the mobility of Cu, Cd, Pb, and Zn may be increased due to the rise in salinity caused by calcium chloride, sodium chloride, and the like [65]. In the embankment-reclaimed saltmarshes, negative correlations between Cr, Cu, Pb, and Zn and salinity imply that seawater intrusion facilitated the mechanisms of these metals’ mobility and repartition [66], whereas the movement and metamorphosis of TMs between distinct phases can be facilitated by high salinity [67]. In both types of reclamation, all TMs had a positive correlation with N (*p* < 0.01). Nitrogen primarily originated in the reclaimed marshes from the heavy use of fertilizers and agrochemicals [68].

**Figure 7:**
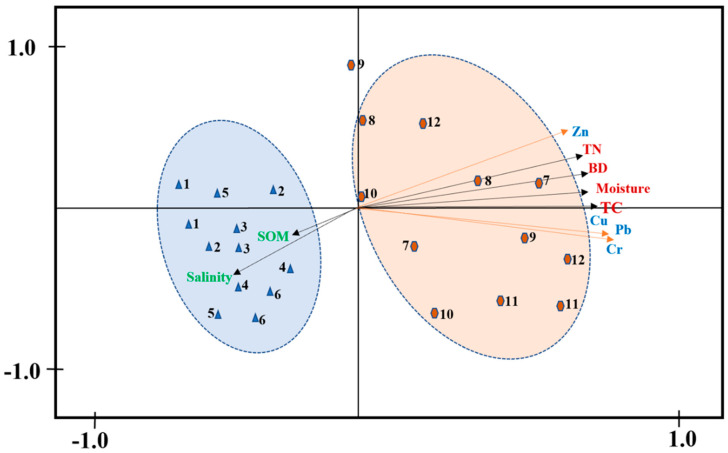
Redundancy analysis (RDA) of TMs, soil properties, and soil samples. Samples 1–6 represent the unreclaimed saltmarshes, and samples 7–12 represent the reclaimed saltmarshes.

The outcome of the RDA analysis is depicted in Figure 7. SOM and salinity were well linked with the first axis, which accounted for 46% of the total variance, and the second axis was largely attributed to moisture and bulk density, explaining 43% of the total variance. Findings also showed the correlations between soil samples from embankment-reclaimed and unreclaimed marshes with various reclamation histories. These soil samples, which were collected at twelve different points, can be grouped into two categories. The correlation between the soil salinity and SOM and the soil samples taken from the unreclaimed marshes suggests that soil salinity and SOM are significant elements in this marsh [69]. In contrast, soil samples taken from the embankment-reclaimed marshes were substantially correlated with Cr, Cu, Pb, Zn moisture, BD, and TN, suggesting that these three variables were the major constraints on the supply, cumulation, absorption, and mobility of these TMs [57].

### 4.2. Identification of Trace Metal Sources

Reclamation has been shown to considerably increase the total amount of TMs in wetlands and the degree of pollution [42,57,70]. The interaction between plant communities and embankments had a significant impact on soil TMs distribution. As shown in Table 6, ANOVA suggests that reclamation depths had substantially greater Cr and Zn concentrations (*p* < 0.01) than Cu and Pb (*p* < 0.01). The average concentrations of Cr, Cu, Pb, and Zn were considerably greater in community reclamation than in community depth and reclamation depth, indicating that reclamation by invasive *S. alterniflora* was a contributing factor in the elevated concentrations of these metals. The content of Cr and Zn was higher than that of Cu and Zn in the community-reclamation-depth measure (*p* < 0.01), which may be connected to the varied irrigation, fertilization, and pesticide application. It can be deduced that soil Cr and Zn were mostly regulated by parent materials when enrichment factors and geographic variation characteristics of these metal levels are taken into consideration [62]. Although community reclamation and community depths had the highest concentrations of Cr and Zn, the differences were not very evident. Therefore, additional research into their sources might be necessary.

**Figure 8:**
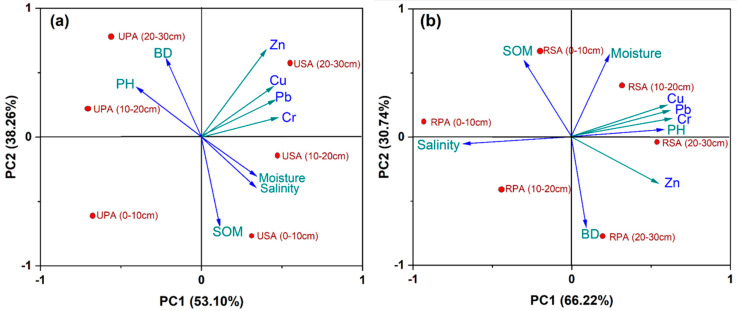
Rotated component matrix and total variance explanation for TM contents in (**a**) the unreclaimed saltmarshes and (**b**) the reclaimed saltmarshes. USA: unreclaimed *S. alteniflora* saltmarsh; RSA: reclaimed *S. alteniflora* saltmarsh; UPA: unreclaimed *P. australis* saltmarsh; RPA: reclaimed *P. australis* saltmarsh.

Figure 8 shows the PCA rotational component matrix and the total variance explained. Principle component (PC, eigenvalue 5.96), explaining 66.22% of the cumulative variance contribution rate, has strong positive loadings in the embankment-reclaimed marshes for Cr (0.86), Cu (0.96), Pb (0.98), and Zn (0.88). While PC2 displayed the gradient variations of PH and BD, PC1 reflected the gradient variations of SOM, salinity, and moisture. In general, Cr, Cu, Pb, and Zn showed significant links with PC1 in the (20–30 cm) depth of the USA, whereas BD and PH showed similar correlations with PC2 (Figure 7a). The principal component (PC, eigenvalue 4.78), which accounts for 49.3% of the total variance in the unreclaimed marshes, is correlated to Cr (0.76), Cu (0.78), Pb (0.81), and Zn (0.78) showing positive loadings. While PC1 displayed the gradient fluctuations of BD, PC2 reflected the gradient variations of SOM, salinity, and moisture. Generally, BD showed substantial relationships with PC2, whereas Cr, Cu, and Pb showed close correlations with PC1 in the 10–20 m depth and Zn in the 20–30 cm depth of the RSA (Figure 8b). PCA analysis has been widely employed by scientists to pinpoint their potential sources as driven by edaphic traits and human activity [44,57]. Due to prior research demonstrating that foreign pollutants were the primary source of As, Cd, Cu, Pb, and Zn, PC1 can be used to represent surface and sub-surface wetland contamination by pesticides, fertilizers, organic livestock wastes, antibiotics, silage effluents, and processed effluents from plantation crops as opposed to PC2 [71,72].

Soil oxidation state and alteration of redox potential in the rhizosphere by invasive *S. alterniflora* and native *P. australis* may have increased SOM, leading to increased Cu and Zn in the reclaimed marshes [73,74], implying that SOM may inhibit TM release rather than TM buildup. However, due to continuous reclamation activities, Cu, Cr, and Zn concentrations in the natural reserve will exceed permitted limits, leading to poor soil quality and considerable damage to the entire coastline [75]. Thus, the BCF values in this study were somewhat low compared to those reported by [61] and [60], as reflected by the *S. alterniflora* saltmarsh, which amassed the highest BCF values when compared to *P. australis*. However, they had the highest quantities of TMs in sediment, as shown in Figure 3. In this context, a comparison of total metal concentrations in soil and plant tissue indicated significant heterogeneity between invasive *S. alterniflora* and native *P. australis*.

The lowest BCF values in high metal-contaminated soil can be attributed to restricted TM uptake by reeds, low metal bioavailability in soils, or both, in the *P. australis* marsh. Nonetheless, TMs may be immobilized in inaccessible forms in wetland soil by binding with organic matter and precipitation or by volatilization and release into the atmosphere under poor conditions [76,77,78]. Considering TM pollution mitigation utilizing phyto-extraction, increased TM buildup in the AGB of plants with BCF and TF values > 1 suggests a larger capacity for metal phyto-extraction from polluted zones [60]. Outcomes of the investigation show that *S. alterniflora* might be classed as a possible Cr, Cu accumulator, and *P. australis* a Zn accumulator, as evidenced by a greater concentration of Cu in leaves and roots, with BCF and TF values (0.63–1.56) and (1.62–2.14) respectively [79,80]. Overall, the *S. alterniflora* marsh cumulated more metals than the *P. australis* marsh. However, relatively low BCF and TF values, especially for Cu and Pb (TF ≤ 0.4) in both reclaimed marshes, suggest that the reclamation seawall embankment altered the ability of wetland marshes to accumulate TM pollution [81] (particularly in the native *P. australis* plant marshes). To regulate and mitigate these pollution sources, relevant departments must adopt suitable policies.

Significant relationships suggest that coastal reclamation regulates the link between TM concentrations and ecological vulnerability in saltmarsh soils [22]. In this investigation, SOM levels in both marshes were negatively linked with the concentrations of the TMs Cr, Cu, Pb, and Zn (*p* < 0.05), indicating that these TMs may have come from external sources [42,82]. The stabilization of Cr and Zn in marsh soils with higher contents of SOM may be the cause of the higher levels of Cr and Zn in both reclaimed saltmarshes [78]. Covelo et al. [83] demonstrated how considerable amounts of Cr and Zn can be bound by soils with high organic matter content. However, increased Cr cumulation in the RSA marsh relative to other sampling sites was primarily caused by the high affinity of Cr for medium organic matter and the historically heavily industrialized nature of most estuary environments [84].

In contrast to the reclaimed marshes, a positive correlation between salinity and TMs was noticed in the unreclaimed marshes (*p* < 0.05), suggesting that a decrease in salinity promotes an increase in the metal uptake by wetland species in natural wetlands compared to reclaimed wetlands [85]. However, in the reclaimed marshes, the apparent negative correlation between the release of Cr, Cu, and Pb and bulk density points to significant retention of Cr, Cu, and Pb in the fine fractions that prevent mobilization by salinization [85]. Because of the narrow pH range, most TMs do not significantly correlate with soil pH [86], but in the reclaimed marshes, there were considerably positive relationships between TMs and PH (Figure 5b). This linkage could be explained by the chemical composition of the sediment metals and the competitiveness between protons and metal ions at the plant-soil-water boundary [87].

### 4.3. Reclamation versus Invasion Context of Trace Metal Dynamics in Coastal Wetlands

In China, invasive *S. alterniflora* and native *P. australis* (and other native wetland plants) have been directly compared in field studies in terms of reclamation and plant invasion [3,16,17,18,19,24,88]. To our knowledge, this is the first study to evaluate the dynamics of TMs in invasive *S. alterniflora* and native *P. australis* saltmarshes relative to invasive plant wetland reclamation and coastal embankment. The invasion of *S. alterniflora* in China has coincided with coastal armoring and the construction of embankments for the reclamation of wetlands and intertidal areas, resulting in the alteration of ecosystem structure and functions despite the derived benefits. As shown in Table 7, reclamation has been proven to dramatically increase the total amount of TMs in wetlands, as well as the degree of pollution, and this differs across native and exotic plant marshes [16,17,18,19].

In this study, coastal embankment reclamation significantly spurred an increase in Cr, Cu, Pb, and Zn levels in both plant marshes according to correlation analysis, indicating synergy between reclamation by embankment and plant invasion. PCA found a factor (1) that accounts for 57% of total variation and is significantly and positively associated with organic matter concentration. The simple correlation and PCA findings imply that the TMs Cr, Cu, Pb, and Zn may have come from various sources and were influenced mainly by anthropogenic pressure [89]. Because translocation factors are all less than one, metals are excluded from aboveground tissues at all sites, irrespective of sediment properties. It has been proposed as a metal resistance mechanism in wetland plants [90], and it is typical of *Spartina* species [91]. *S. alterniflora* has been found with TFs < 1 in saltmarshes in the United States [79] and Argentina [92,93]. Moreover, the potential of wetland plant marshes to accumulate TM contamination was affected by the reclamation seawall, as reflected in the low TF values (TF < 1) obtained. Overall, the findings of studies in China may not be reflective of saltmarshes in other regions, because the functions of invasive and native species are likely reversed. Furthermore, differences in plant phenology, root-influenced element availability/transport, reclamation type, invasion dynamics, and climatic factors all influence outcomes.

## 5. Conclusions

Due to multiple anthropogenic pollution sources, the reclaimed saltmarshes had significantly higher levels of all examined TMs than the unreclaimed saltmarshes. Invasion by *S. alterniflora* and the reclamation by seawall embankments can both effectively intercept and retain heavy metal contaminants, restricting the transit of exogenous metal pollutants to coastal habitats. Additionally, the significant environmental variables influencing the dispersion and buildup of TMs are organic matter and soil grain size. According to international sediment quality guidelines SQGs, we discovered that Cr, Cu, and Zn in the saltmarshes, particularly in the reclaimed regions, have caused a moderately polluted ecological risk. The soils from marshes on the coastline of the Dongtai wetlands were moderately to severely contaminated by Cu, Zn, and Cr based on I_geo_ risk indices and the findings of previous research. Overall, soils impacted by reclamation posed a greater ecological risk than unreclaimed soils. The distribution of TM components, particularly Cr and Zn, has shifted as a result of reclamation by seawall embankment. Due to its high-risk assessment code values, Cr posed a moderate-to-high potential ecological threat for the saltmarshes, whereas Pb demonstrated low potential ecological concerns. Although the risk threshold for Cu and Pb contamination in soil is somewhat low, consideration should be given to the risk of Cu pollution in saltmarshes because of its particularly high acid-replaceable contents and significant cumulation levels compared to background values. In addition to highlighting the positive influence of *S. alterniflora* on the mobility of exogenous toxic metals to the sea, these findings provide a novel and comprehensive insight into the thresholds, sources, and outcomes of heavy metal pollution in the saltmarsh ecosystem of seawall embankment-reclaimed and unreclaimed soils of invasive and native coastal saltmarshes.

### Management Implications and Perspective

This study determined that coastal embankment reclamation had significant effects on TMs dynamics in the invasive *S. alterniflora* and native *P. australis* saltmarshes. *S. alterniflora* was observed to discharge more metals into the marsh ecosystem by excretion and leaf accumulation than Phragmites australis. When *P. australis* is replaced with invasive *S. alterniflora*, metal bioavailability would be diminished since *P. australis* moves more metal load into readily degradable leaf tissues (as opposed to refractory stems, roots, and rhizomes). The significant increase in TM contents in the coastal embankment-reclaimed saltmarsh of both plant species could be attributed to lower soil-plant litter input and lower soil salinity and moisture levels. Based on statistical analysis, our findings suggest that a synergy between embankment seawall reclamation and *S. alterniflora* invasion had a profound impact on *P. australis* salt marsh, despite *S. alterniflora* having the most impact.

In the saltmarshes of the study region, coastal embankment reclamation and the replacement of native *P. australis* by invasive *S. alterniflora* significantly weakened the saltmarshes and simultaneously spurred TM cumulation, as well as potentially limiting the capacity of marsh plants to accumulate TMs. As evidenced in this study, seawall construction and the introduction of IAPS can have negative consequences, such as diminished TMs cumulation, resulting in increased soil TM contamination, among other causes. Reclamation by the coastal embankment and IAPS could reduce regionally distributed biodiversity, disrupt ecological processes and services, and jeopardize the long-term viability of coastal wetlands [96]. Coastal embankment reclamation, on the other hand, could effectively curb *S. alterniflora* from invasion [24], provide a buffer stratum that significantly lessens flooding and storms [97], and safeguards native saltmarshes, fostering a minimal loss of ecological functions and benefits [98]. These scientific findings provide stakeholders and policymakers with leverage to reconcile seawall reclamation and exotic plant expansion and allowmore investigation into the synergistic impacts of tradeoffs between alien plant invasion and reclamation embankments in coastal wetlands.

## Figures and Tables

**Table 1:** Soil physical and chemical properties (mean ± SE, *n* = 12) in the USA, RSA, UPA, and RPA saltmarshes. Within the same area and at the same depth of soil, lower-case superscript letters indicate statistical significance between the unreclaimed and sea embankment-reclaimed saltmarshes.

	Depth (cm)	Moisture (%)	BD (g cm^−3^)	Salinity (%)	PH	SOM (g kg^−1^)
USA	0–10	63.86 ± 1.36 ^b^	0.89 ± 0.04 ^a^	1.76 ± 0.05 ^a^	8.53 ± 0.05 ^a^	33.04 ± 1.48 ^a^
	10–20	51.20 ± 2.04 ^b^	0.97 ± 0.02 ^a^	1.53 ± 0.04 ^a^	8.47 ± 0.08 ^a^	27.43 ± 0.96 ^b^
	20–30	44.61 ± 1.24 ^a^	1.32 ± 0.05 ^b^	1.41 ± 0.05 ^a^	8.37 ± 0.04 ^a^	21.49 ± 0.86 ^b^
RSA	0–10	35.66 ± 1.46 ^b^	1.13 ± 0.05 ^a^	0.67 ± 0.03 ^b^	9.04 ± 0.03 ^a^	18.75 ± 0.34 ^a^
	10–20	28.80 ± 1.05 ^a^	1.27 ± 0.10 ^a^	0.52 ± 0.03 ^b^	9.18 ± 0.03 ^a^	13.59 ± 3.26 ^a^
	20–30	25.94 ± 0.80 ^a^	1.33 ± 0.12 ^a^	0.47 ± 0.02 ^b^	9.26 ± 0.05 ^a^	10.11 ± 2.22 ^a^
UPA	0–10	21.07 ± 1.14 ^a^	0.88 ± 0.08 ^a^	0.97 ± 0.08 ^a^	8.47 ± 0.07 ^a^	28.44 ± 1.05 ^a^
	10–20	22.34 ± 1.08 ^a^	1.53 ± 0.08 ^b^	0.93 ± 0.05 ^a^	8.66 ± 0.05 ^a^	22.43 ± 0.88 ^a^
	20–30	22.22 ± 0.93 ^a^	1.57 ± 0.10 ^b^	0.84 ± 0.05 ^a^	8.89 ± 0.07 ^a^	18.66 ± 0.87 ^b^
RPA	0–10	17.56 ± 0.78 ^b^	1.18 ± 0.08 ^a^	0.73 ± 0.02 ^a^	8.91 ± 0.03 ^a^	15.63 ± 4.10 ^a^
	10–20	12.45 ± 1.25 ^a^	1.56 ± 0.09 ^a^	0.69 ± 0.02 ^a^	8.98 ± 0.03 ^a^	12.15 ± 4.06 ^a^
	20–30	10.55 ± 0.87 ^a^	1.58 ± 0.16 ^b^	0.64 ± 0.01 ^a^	9.06 ± 0.02 ^a^	8.62 ± 3.36 ^b^

**Table 2:** The concentrations of trace metals (TMs) in soil (mean ± SE, *n* = 12) in the USA, RSA, UPA, and RPA saltmarshes. Within the same area and at the same depth of soil, lower-case superscript letters indicate statistical significance (*p* < 0.05) between the unreclaimed and sea embankment-reclaimed saltmarshes.

	Depth (cm)	Cr (mg kg^−1^)	Cu (mg kg^−1^)	Pb (mg kg^−1^)	Zn (mg kg^−1^)
USA	0–10	50.77 ± 0.59 ^a^	12.32 ± 0.42 ^a^	13.33 ± 0.58 ^a^	40.81 ± 0.25 ^a^
	10–20	67.89 ± 0.54 ^a^	16.32 ± 0.58 ^a^	18.65 ± 0.87 ^a^	53.68 ± 0.66 ^a^
	20–30	80.48 ± 0.21 ^a^	23.29 ± 0.48 ^a^	23.82 ± 0.68 ^a^	63.62 ± 0.75 ^a^
RSA	0–10	65.25 ± 0.28 ^a^	21.39 ± 0.55 ^b^	18.45 ± 0.44 ^a^	56.29 ± 0.48 ^a^
	10–20	84.05 ± 0.77 ^a^	26.27 ± 0.58 ^b^	24.53 ± 0.27 ^a^	65.58 ± 0.98 ^a^
	20–30	112.65 ± 0.84 ^b^	32.56 ± 0.12 ^b^	32.34 ± 0.84 ^b^	90.69 ± 0.14 ^b^
UPA	0–10	22.55 ± 0.87 ^a^	7.52 ± 0.54 ^a^	6.25 ± 0.28 ^a^	33.47 ± 0.02 ^a^
	10–20	33.04 ± 0.32 ^a^	8.86 ± 0.58 ^a^	8.63 ± 0.58 ^a^	44.21 ± 0.96 ^a^
	20–30	41.98 ± 0.54 ^a^	12.45 ± 0.19 ^a^	13.43 ± 0.89 ^a^	55.97 ± 0.33 ^a^
RPA	0–10	37.87 ± 0.54 ^b^	11.93 ± 0.05 ^b^	9.21 ± 0.96 ^a^	51.70 ± 0.87 ^b^
	10–20	51.08 ± 0.45 ^b^	14.73 ± 0.26 ^b^	13.20 ± 0.87 ^a^	60.74 ± 0.05 ^a^
	20–30	66.75 ± 0.39 ^b^	18.22 ± 0.45 ^a^	19.62 ± 0.99 ^a^	78.71 ± 0.95 ^a^

**Table 3:** Sediment quality guidelines SQG (mg kg^−1^) in different regions. CB: Consensus Based; TEC: Threshold Effect Concentration; SEPA: State Environmental Protection Administration; ISQV: Interim Sediment Quality Value; NOAA: National Oceanic and Atmospheric Administration; SQAV: Sediment Quality Advisory Value; US EPA: United States Environmental Protection Agency; MSQG: Marine Sediment Quality Guideline; LEL: Lowest effect level; SEL: severe effect level; TEL: Threshold Effect level.

SQG		Cr	Cu	Pb	Zn	Reference
CB	TEC	43.4	31.6	35.8	121	[47]
China SEPA	MSQG	80	35	60	150	[48]
Hong kong ISQVs	ISQV-low	80	65	75	200	[49]
	ISQV-high	370	270	218	410	[49]
NOAA	ERL	81	34	46.7	150	[50]
Ontario guidelines	LEL	26	16	31	120	[51]
	SEL	110	110	250	820	[51]
SQAV	TEL	36	28	37	98	[52]
SQO Netherlands	Target	-	36	85	140	[53]
US EPA	Non-polluted	<25	<25	<40	<90	[54]
	Moderate-polluted	25–75	25–50	40–60	90–200	[54]
	Heavily-polluted	>75	>50	>60	>200	[54]

**Table 4:** Mean TM concentrations (mg kg^−1^) in soils from the Dongtai Estuary and other relevant wetland habitats in other regions.

Location	Cr	Cu	Pb	Zn	References
Yangtze River Delta China	68.3	13	16.6	81.2	[22]
Yangtze River Estuary, China	69.4	38.4	28.2	99.5	[55]
Tidal flat of Jiangsu, China	51.4	15.68	11.77	51.62	[56]
Pearl River Delta, China	-	70.0	48.3	156.8	[57]
Kavak Delta, Turkey	163.0	37.5	26.5	84.2	[59]
Langat River, Malaysia	2.68	-	15.5	29.7	[58]
Dongtai estuary, Jiangsu China	112.6	32.6	32.3	90.7	This study

**Table 5:** Average TM concentration in *S. alterniflora* and *P. australis* biomass from saltmarsh plants under various forms of reclamation.

Saltmarsh	Biomass	Trace Metals (mg kg^−1^)			
		Cr	Cu	Pb	Zn
USA	Leaves	30.86	13.46	7.63	37.52
	Stem	10.31	7.12	3.00	19.45
	Roots	13.69	26.62	7.05	56.81
RSA	Leaves	18.68	4.02	5.35	21.84
	Stem	7.19	7.81	2.43	13.34
	Roots	52.04	17.52	18.4	51.17
UPA	Leaves	15.94	8.40	4.00	36.31
	Stem	6.23	5.71	3.17	24.92
	Roots	3.87	13.3	3.00	47.61
RPA	Leaves	5.73	2.29	2.96	26.41
	Stem	4.14	4.35	3.22	18.33
	Roots	26.36	10.25	3.82	28.81

**Table 6:** Three-way ANOVA analysis to determine the statistical significance of the impacts of species, reclamation, depth, and their interconnections on soil heavy metal fractions.

Variation Source	Cr (mg kg^−1^)	Cu (mg kg^−1^)	Pb (mg kg^−1^)	Zn (mg kg^−1^)
Community	2312.13 ***	625.43 ***	660.08 ***	1858.06 ***
Reclamation	961.52 ***	277.58 ***	289.57 ***	775.22 ***
Depth	691.94 ***	202.87 ***	210.62 ***	564.23 ***
Community × Reclamation	1340.02 ***	362.47 ***	382.56 ***	1076.86 ***
Community × Depth	1046.02 ***	282.94 ***	298.62 ***	840.59 ***
Reclamation × Depth	670.32 **	181.31 **	191.36 **	538.65 **
Community × Reclamation × Depth	229.39 **	62.04 **	65.48 **	184.33 **

** *p* < 0.01, *** *p* < 0.001.

**Table 7:** Reclamation versus invasion context of TM cycling and their impacts across coastal wetlands across the world.

Location	Reclamation Type	Invasion Type	Trace Metals	Reclamation/Invasion Effect	References
United States of America	-	Phragmites australis invasion	Cr, Cu, Hg, Pb, Zn	Significantly negative on (Cu and Zn)	[79]
China	Unspecified wetland reclamation	-	Cd, Cr, Cu, Ni, Pb, and Zn	Significantly negative on (Cd, Cr, Cu, Ni, Pb, and Zn)	[94]
China	Agriculturalland conversion	-	Fe, Mn, Cd, Cr, Cu, Ni, Pb, and Zn	Significantly negative on (Cd, Cu, Pb, and Zn)	[42]
China	Unspecified wetland reclamation	-	Al, Fe, Mn, Cu, Cr, Ni, Zn, and Pb	Significantly negative on (Cu, Cr, Zn, and Pb)	[95]
China	Agricultural land conversion	-	As, Cd, Zn, Cu, and Pb	Significantly negative on (As, Cd, Zn, Cu, and Pb)	[57]
China	Unspecified wetland reclamation	Spartina alterniflora invasion	Fe, Mn, Al, Cu, Zn, Cd, Pb, Cr, Ni, and As	Significantly negative on Cd, Pb, Zn, and Ni	[22]
China	Sea embankment	Spartina alterniflora invasion	Cr, Cu, Pb, and Zn	Significantly negative on (Cr, Cu, Pb, and Zn)	This study

## Data Availability

Not applicable.
